# Supplementary material for: DyME: An MD-based engine exploiting HTP mutagenesis for protein engineering and recognition mimicry
Source: PLoS Comput Biol. 2026 Oct 7;22(10):e1014221. doi: 10.1371/journal.pcbi.1014221 (PMC13671646; doi:10.1371/journal.pcbi.1014221)
Supplement: Supplementary file 1 [file pcbi.1014221.s001.docx]

**S1 Appendix – Software Dependencies**

**Table A – Dependencies of the DyME API (Anaconda packages)**

| **Package** | **Version** |  | **Package** | **Version** |  | **Package** | **Version** |
| --- | --- | --- | --- | --- | --- | --- | --- |
| _libgcc_mutex | 0.1 |  | brotlipy | 0.7.0 |  | entrypoints | 0.4 |
| _openmp_mutex | 5.1 |  | bzip2 | 1.0.8 |  | expat | 2.4.9 |
| _tflow_select | 2.3.0 |  | c-ares | 1.18.1 |  | fftw | 3.3.10 |
| abseil-cpp | 20210324.2 |  | c-compiler | 1.3.0 |  | filelock | 3.9.0 |
| absl-py | 1.3.0 |  | ca-certificates | 2022.9.24 |  | flake8 | 4.0.1 |
| aiohttp | 3.8.1 |  | cached-property | 1.5.2 |  | flask | 2.2.2 |
| aiosignal | 1.3.1 |  | cached_property | 1.5.2 |  | flatbuffers | 2.0.6 |
| alabaster | 0.7.12 |  | cachetools | 4.2.4 |  | flit-core | 3.6.0 |
| ambertools | 22.0 |  | certifi | 2022.9.24 |  | fontconfig | 2.13.96 |
| appdirs | 1.4.4 |  | cffi | 1.15.1 |  | fonttools | 4.28.5 |
| argh | 0.26.2 |  | cftime | 1.5.1.1 |  | fortran-compiler | 1.3.0 |
| arpack | 3.7.0 |  | chardet | 4.0.0 |  | freetype | 2.11.0 |
| arrow | 1.2.3 |  | charset-normalizer | 2.0.10 |  | frozenlist | 1.3.0 |
| astor | 0.8.1 |  | click | 8.0.4 |  | gast | 0.4.0 |
| astroid | 2.14.2 |  | cloudpickle | 2.0.0 |  | gcc | 9.4.0 |
| astunparse | 1.6.3 |  | colorama | 0.4.6 |  | gcc_impl_linux-64 | 9.4.0 |
| async-timeout | 4.0.2 |  | colorful | 0.5.4 |  | gcc_linux-64 | 9.4.0 |
| atomicwrites | 1.4.0 |  | comm | 0.1.2 |  | gettext | 0.19.8.1 |
| attrs | 22.1.0 |  | compilers | 1.3.0 |  | gfortran | 9.4.0 |
| autopep8 | 1.6.0 |  | conda | 22.9.0 |  | gfortran_impl_linux-64 | 9.4.0 |
| babel | 2.11.0 |  | conda-build | 3.23.0 |  | gfortran_linux-64 | 9.4.0 |
| backcall | 0.2.0 |  | conda-package-handling | 2.0.2 |  | giflib | 5.2.1 |
| backports | 1.1 |  | conda-package-streaming | 0.7.0 |  | glib | 2.68.4 |
| backports.functools | 1.6.4 |  | contourpy | 1.0.5 |  | glib-tools | 2.68.4 |
| beautifulsoup4 | 4.11.1 |  | cookiecutter | 1.7.3 |  | glob2 | 0.7 |
| binaryornot | 0.4.4 |  | cryptography | 39.0.1 |  | google-auth | 1.35.0 |
| binutils | 2.36.1 |  | cudatoolkit | 10.2.89 |  | google-auth-oauthlib | 0.4.6 |
| binutils_impl_linux-64 | 2.36.1 |  | curl | 7.82.0 |  | google-pasta | 0.2.0 |
| binutils_linux-64 | 2.36 |  | cxx-compiler | 1.3.0 |  | griddataformats | 0.6.0 |
| biopython | 1.79 |  | cycler | 0.11.0 |  | grpcio | 1.46.1 |
| black | 22.6.0 |  | cython | 0.29.25 |  | gsd | 2.5.1 |
| blas | 1.0 |  | dbus | 1.13.18 |  | gst-plugins-base | 1.18.4 |
| bleach | 4.1.0 |  | debugpy | 1.5.1 |  | gstreamer | 1.18.5 |
| blinker | 1.5 |  | decorator | 5.1.1 |  | gxx | 9.4.0 |
| blosc | 1.21.0 |  | defusedxml | 0.7.1 |  | gxx_impl_linux-64 | 9.4.0 |
| boost-cpp | 1.74.0 |  | diff-match-patch | 20200713 |  | gxx_linux-64 | 9.4.0 |
| bottleneck | 1.3.5 |  | dill | 0.3.6 |  | h5py | 3.6.0 |
| brotli | 1.0.9 |  | dnspython | 2.2.1 |  | hdf4 | 4.2.15 |
| brotli-bin | 1.0.9 |  | docutils | 0.18.1 |  | hdf5 | 1.12.1 |
| hdf5-1107 | 1.10.7 |  | libevent | 2.1.10 |  | modeller | 10.4 |
| hypothesis | 6.36.2 |  | libffi | 3.3 |  | mpi | 1.0 |
| icu | 68.2 |  | libgcc-devel_linux-64 | 9.4.0 |  | mpi4py | 3.1.3 |
| idna | 3.4 |  | libgcc-ng | 11.2.0 |  | msgpack-python | 1.0.3 |
| imagesize | 1.4.1 |  | libgfortran-ng | 11.2.0 |  | multidict | 6.0.2 |
| importlib-metadata | 4.11.3 |  | libgfortran5 | 11.2.0 |  | multiprocess | 0.70.12.2 |
| importlib_metadata | 4.11.3 |  | libglib | 2.68.4 |  | munkres | 1.1.4 |
| importlib_resources | 5.10.0 |  | libgomp | 11.2.0 |  | mypy_extensions | 0.4.3 |
| inflection | 0.5.1 |  | libiconv | 1.17 |  | mysql-common | 8.0.28 |
| iniconfig | 1.1.1 |  | liblapack | 3.9.0 |  | mysql-libs | 8.0.28 |
| intel-openmp | 2021.4.0 |  | liblief | 0.12.3 |  | nbclient | 0.5.13 |
| intervaltree | 3.1.0 |  | libllvm11 | 11.1.0 |  | nbconvert | 6.4.4 |
| ipykernel | 6.19.2 |  | libnetcdf | 4.8.1 |  | nbformat | 5.7.0 |
| ipython | 7.32.0 |  | libnghttp2 | 1.47.0 |  | ncurses | 6.3 |
| ipython_genutils | 0.2.0 |  | libnsl | 2.0.0 |  | nest-asyncio | 1.5.6 |
| ipywidgets | 8.0.2 |  | libpng | 1.6.37 |  | netcdf-fortran | 4.5.4 |
| isort | 5.10.1 |  | libpq | 13.5 |  | netcdf4 | 1.6.2 |
| itsdangerous | 2.0.1 |  | libprotobuf | 3.14.0 |  | networkx | 2.8.4 |
| jbig | 2.1 |  | libsanitizer | 9.4.0 |  | nglview | 3.0.3 |
| jedi | 0.18.1 |  | libsodium | 1.0.18 |  | nspr | 4.32 |
| jeepney | 0.7.1 |  | libspatialindex | 1.9.3 |  | nss | 3.77 |
| jellyfish | 0.9.0 |  | libssh2 | 1.10.0 |  | numexpr | 2.8.4 |
| jinja2 | 3.1.2 |  | libstdcxx-devel_linux-64 | 9.4.0 |  | numpy | 1.23.5 |
| jinja2-time | 0.2.0 |  | libstdcxx-ng | 11.2.0 |  | numpy-base | 1.23.5 |
| joblib | 1.1.1 |  | libtiff | 4.3.0 |  | numpydoc | 1.5.0 |
| jpeg | 9e |  | libuuid | 2.32.1 |  | oauthlib | 3.2.2 |
| jsonschema | 4.17.3 |  | libwebp | 1.2.4 |  | ocl-icd | 2.3.1 |
| jupyter_client | 7.4.9 |  | libwebp-base | 1.2.4 |  | ocl-icd-system | 1.0.0 |
| jupyter_core | 5.2.0 |  | libxcb | 1.13 |  | openmm | 7.7.0 |
| jupyterlab_pygments | 0.1.2 |  | libxkbcommon | 1.0.3 |  | openmm-setup | 1.3 |
| jupyterlab_widgets | 3.0.3 |  | libxml2 | 2.9.12 |  | openmpi | 4.1.2 |
| keras | 2.6.0 |  | libzip | 1.8.0 |  | openssl | 1.1.1u |
| keras-preprocessing | 1.1.2 |  | libzlib | 1.2.11 |  | opt_einsum | 3.3.0 |
| kernel-headers_linux-64 | 2.6.32 |  | lz4-c | 1.9.3 |  | packaging | 22.0 |
| keyring | 23.4.0 |  | lzo | 2.10 |  | packmol | 20010 |
| kiwisolver | 1.4.4 |  | marKdown | 3.4.1 |  | pandas | 1.5.3 |
| krb5 | 1.19.4 |  | markupsafe | 2.1.1 |  | pandocfilters | 1.5.0 |
| lazy-object-proxy | 1.7.1 |  | matplotlib-base | 3.7.0 |  | parmed | 3.4.3 |
| lcms2 | 2.12 |  | matplotlib-inline | 0.1.6 |  | parso | 0.8.3 |
| ld_impl_linux-64 | 2.36.1 |  | mccabe | 0.6.1 |  | patch | 2.7.6 |
| lerc | 3.0 |  | mdanalysis | 2.0.0 |  | patchelf | 0.17.2 |
| libarchive | 3.5.2 |  | mdanalysistests | 2.0.0 |  | pathspec | 0.10.3 |
| libblas | 3.9.0 |  | mdtraj | 1.9.7 |  | patsy | 0.5.3 |
| libbrotlicommon | 1.0.9 |  | mistune | 0.8.4 |  | pcre | 8.45 |
| libbrotlidec | 1.0.9 |  | mkl | 2021.4.0 |  | peewee | 3.14.10 |
| libbrotlienc | 1.0.9 |  | mkl-service | 2.4.0 |  | perl | 5.32.1 |
| libclang | 11.1.0 |  | mkl_fft | 1.3.1 |  | pexpect | 4.8.0 |
| libcurl | 7.82.0 |  | mkl_random | 1.2.2 |  | pickleshare | 0.7.5 |
| libdeflate | 1.8 |  | mmtf-python | 1.1.2 |  | pika | 1.3.0 |
| libedit | 3.1.20221030 |  | mock | 4.0.3 |  | pillow | 9.0.1 |
| libev | 4.33 |  | mod_wsgi | 4.9.1 |  | pip | 22.3.1 |
| pkginfo | 1.8.3 |  | pytables | 3.7.0 |  | scikit-learn | 1.2.1 |
| platformdirs | 2.5.2 |  | pytest | 7.1.2 |  | scipy | 1.10.0 |
| pluggy | 1.0.0 |  | python | 3.9.12 |  | seaborn | 0.12.2 |
| pooch | 1.4.0 |  | python-dateutil | 2.8.2 |  | setuptools | 65.6.3 |
| poyo | 0.5.0 |  | python-fastjsonschema | 2.16.2 |  | sip | 4.19.13 |
| prettyprinter | 0.18.0 |  | python-flatbuffers | 1.12 |  | six | 1.16.0 |
| prompt-toolkit | 3.0.36 |  | python-libarchive-c | 3.2 |  | snappy | 1.1.9 |
| protobuf | 3.14.0 |  | python-lsp-black | 1.1.0 |  | snowballstemmer | 2.2.0 |
| psutil | 5.9.0 |  | python-lsp-jsonrpc | 1.0.0 |  | sortedcontainers | 2.4.0 |
| ptyprocess | 0.7.0 |  | python-lsp-server | 1.3.3 |  | soupsieve | 2.3.2.post1 |
| py | 1.11.0 |  | python-slugify | 6.1.1 |  | sphinx | 5.0.2 |
| py-lief | 0.12.3 |  | python_abi | 3.9 |  | sphinxcontrib-applehelp | 1.0.2 |
| pyasn1 | 0.4.8 |  | pytoolconfig | 1.2.5 |  | sphinxcontrib-devhelp | 1.0.2 |
| pyasn1-modules | 0.2.7 |  | pytz | 2022.7 |  | sphinxcontrib-htmlhelp | 2.0.0 |
| pycodestyle | 2.8.0 |  | pyu2f | 0.1.5 |  | sphinxcontrib-jsmath | 1.0.1 |
| pycosat | 0.6.4 |  | pyxdg | 0.27 |  | sphinxcontrib-qthelp | 1.0.3 |
| pycparser | 2.21 |  | pyyaml | 6.0 |  | sqlite | 3.38.2 |
| pydocstyle | 6.3.0 |  | pyzmq | 23.2.0 |  | statsmodels | 0.13.2 |
| pyflakes | 2.4.0 |  | qdarkstyle | 3.0.2 |  | sysroot_linux-64 | 2.12 |
| pygments | 2.11.2 |  | qstylizer | 0.2.2 |  | tensorboard | 2.6.0 |
| pyjwt | 2.6.0 |  | readline | 8.2 |  | tensorboard-data-server | 0.6.0 |
| pylint | 2.16.2 |  | requests | 2.28.1 |  | tensorboard-plugin-wit | 1.8.1 |
| pymongo | 4.1.1 |  | requests-oauthlib | 1.3.1 |  | tensorflow | 2.6.0 |
| pyopenssl | 23.0.0 |  | ripgrep | 13.0.0 |  | tensorflow-base | 2.6.0 |
| pyparsing | 3.0.9 |  | rope | 1.7.0 |  | tensorflow-estimator | 2.6.0 |
| pyqt | 5.12.3 |  | rsa | 4.9 |  | termcolor | 2.1.0 |
| pyrsistent | 0.18.1 |  | rtree | 1.0.1 |  | testpath | 0.6.0 |
| pysocks | 1.7.1 |  | ruamel_yaml | 0.15.100 |  | text-unidecode | 1.3 |
| secretstorage | 3.3.1 |  | tqdm | 4.64.1 |  | textdistance | 4.2.2 |
| toml | 0.10.2 |  | traitlets | 5.7.1 |  | threadpoolctl | 3.0.0 |
| tomli | 2.0.1 |  | typing-extensions | 4.4.0 |  | three-merge | 0.1.1 |
| tomlkit | 0.11.1 |  | typing_extensions | 4.4.0 |  | tinycss2 | 1.2.1 |
| toolz | 0.12.0 |  | tzdata | 2022g |  | tk | 8.6.11 |
| tornado | 6.2 |  | ucx | 1.9.0 |  | xorg-xproto | 7.0.31 |
| werkzeug | 2.2.2 |  | ujson | 5.4.0 |  | xz | 5.2.10 |
| wheel | 0.35.1 |  | unidecode | 1.3.3 |  | yaml | 0.2.5 |
| widgetsnbextension | 4.0.3 |  | urllib3 | 1.26.14 |  | yapf | 0.32.0 |
| wrapt | 1.14.1 |  | watchdog | 2.1.6 |  | yarl | 1.7.2 |
| wurlitzer | 3.0.2 |  | wcwidth | 0.2.5 |  | zeromq | 4.3.4 |
| xorg-kbproto | 1.0.7 |  | webencodings | 0.5.1 |  | zipp | 3.11.0 |
| xorg-libice | 1.0.10 |  | xorg-libx11 | 1.7.2 |  | zlib | 1.2.11 |
| xorg-libsm | 1.2.3 |  | xorg-libxext | 1.3.4 |  | zstandard | 0.19.0 |
| zstd | 1.5.1 |  | xorg-libxt | 1.2.1 |  | xorg-xextproto | 7.3.0 |

**PIP Packages**

| **Package** | **Version** |  | **Package** | **Version** |  | **Package** | **Version** |
| --- | --- | --- | --- | --- | --- | --- | --- |
| amberlite | 22 |  | nose | 1.3.7 |  | ruamel-yaml-clib | 0.2.7 |
| amberutils | 21 |  | packmol-memgen | 1.2.3rc0 |  | sander | 22 |
| jc | 1.23.0 |  | pdb4amber | 22 |  | weblogo | 3.7.12 |
| logomaker | 0,8 |  | pdbfixer | 1.8.1 |  | xgboost | 2.0.3 |
| mmpbsa-py | 16 |  | pytraj | 2.0.6 |  | xmltodict | 0.13.0 |
| natsort | 8.4.0 |  | ruamel-yaml | 0.17.21 |  |  |  |

**DyME Operating Environment Considerations**

- DyME assumes that servers acting as “MD nodes” are setup with up-to-date NVIDIA drivers, the CUDA toolkit version >11, and GPU cards set on EXCLUSIVE_PROCESS mode: so that each simulation runs exclusively on one GPU card.
- Servers acting as “Scavenger nodes” are expected to have at least 8 CPU cores. This is hardcoded in generated MPI commands, so that energy calculations and water mapping can be parallelized and performed efficiently.
- For large-scale node deployments, existing computing clusters (*e.g*. HPC) with queueing systems are recommended. However, DyME nodes account for manual execution of worker nodes without requiring a queue manager. Given the diversity of queue managers and their unique configurations, node deployment must be handled by the user.
- Nodes have been conveniently built into containers, using docker and apptainer. See DyME install guidelines for more information.

**Fig A. The DyME internal MongoDB Database Structure**
