# Supplementary material for: DyME: An MD-based engine exploiting HTP mutagenesis for protein engineering and recognition mimicry
Source: PLoS Comput Biol. 2026 Oct 7;22(10):e1014221. doi: 10.1371/journal.pcbi.1014221 (PMC13671646; doi:10.1371/journal.pcbi.1014221)
Supplement: Supplementary file 2 [file pcbi.1014221.s002.docx]

**S2 Appendix - Details on Methods and Implementations**

**Text A - Automated mutant library definition and structural generation**

This process, as explained in Fig A, runs automatically at the backend after completing the project creation wizard. All singlet, doublet and triplet mutation combinations for each cluster of anchor points are computed (Fig A1) with the ‘*combinations’* class of the Python package *itertools,* producing a list of “*mutational combinations*” (Fig A2). Redundant mutant records are prevented by making use of a symmetric difference operator. Each resulting combination is inserted as an individual database record, representing one unique mutant.

Mutant records are made unique by assigning them with an incremental ID and their parent project ID. Each of these records contains a field called “*mutant*” (highlighted in a red box in Fig A3), which indicates its corresponding mutations. A field named “*status*” determines the state of the mutant throughout the workflow, which can indicate “pending”, “processing”, “ready_to_scavenge”, “scavenging”, “done” or “failed”.

**Fig A. Mutant library construction process.** The mutational matrix (1) is iterated using a combinatorial operator, which produces a non-redundant list of mutational combinations (2). Each mutant is stored as a unique Mutant Database Record indicating its corresponding point mutations (3).

**Text B - The “DyME Database Reporter” module for OpenMM**

The in-house developed OpenMM extension “DyME Database Reporter” periodically reports the status of running simulations into the central DB. To do so, it updates the field “status_vars” of the corresponding mutant record, indicating which node and GPU card is running the simulation, the elapsed and remaining computing time, the number of steps simulated, and several more fields relevant to the process. The *“TXT OpenMM reporter”* was used as template to build this component, following existing documentation provided by Eastman *et al* [1].

**Text C - Computing Binding Free Energy Calculations**

DyME uses MMPBSA.py [2] to calculate total binding free energies, per-residue and pairwise energy decompositions. The configuration templates required to run this tool for any given simulation are generated internally. Each energy scheme (per-residue and pairwise) generates two output files in CSV format. One such file contains the total binding energy of the complex, and the remaining file contains energy decomposition records. The “DELTAS” table within this file is extracted into a *pandas* dataframe [3] for re-formatting and further preparation before database insertion. All per-residue energy decomposition values are stored, whereas pairwise energy values are only stored for the positions defined as anchor points. DyME optimizes MMPBSA.py [2] by purposely sourcing MD trajectory files from an internal *ramdrive* partition. This results in IOPS occurring 2 orders of magnitude faster than sourcing from disk, which dramatically reduces execution times.

**Text D - Computing Atomic Contact Extraction (Forward and Reverse)**

DyME uses the *TrajectoryIterator* object and *search_hbonds*() function of PYTRAJ [4] (the Python front-end for the AmberTools [5] package CPPTRAJ [6]) to compute forward and reverse inter-molecular contacts. A 135° angle and a 3.5 Å distance cutoff are used by default. The “acceptor” and “donor” residue masks are calculated automatically by DyME (using the project’s *“molecular object”* definitions). The output of this function is converted to Python dictionaries containing atomic contact data. Contact frequency thresholds of 10%, 20% and 50% are extracted, classified and stored.

**Text E - Computing RMSD and Best MD Frame**

The RMSD of the MD trajectories is computed using the function *rmsd*() of MDTraj [7][31]. A single array of values is produced, each position representing the mean deviation at each MD frame.

The potential energy of the system at each frame exists within the HDF5 trajectory file. The “best frame” of the trajectory is assumed to be that of lowest energy value. This pose is extracted, stored in PDB format and used for comparative structural superposition in the web-GUI.

**References**

1.	Eastman P, Swails J, Chodera JD, McGibbon RT, Zhao Y, Beauchamp KA, et al. OpenMM 7: Rapid development of high performance algorithms for molecular dynamics. Gentleman R, editor. PLOS Comput Biol. 2017;13: e1005659. doi:10.1371/journal.pcbi.1005659

2.	Miller BR, McGee TD, Swails JM, Homeyer N, Gohlke H, Roitberg AE. *MMPBSA.py* : An Efficient Program for End-State Free Energy Calculations. J Chem Theory Comput. 2012;8: 3314–3321. doi:10.1021/ct300418h

3.	McKinney W, others. Data structures for statistical computing in python. Proceedings of the 9th Python in Science Conference. Austin, TX; 2010. pp. 51–56.

4.	Nguyen H, Swails J, Roe DR, Cody. Amber-MD/pytraj: v2.0.0. Zenodo; 2017. doi:10.5281/zenodo.439134

5.	Case DA, Aktulga HM, Belfon K, Cerutti DS, Cisneros GA, Cruzeiro VWD, et al. AmberTools. J Chem Inf Model. 2023;63: 6183–6191. doi:10.1021/acs.jcim.3c01153

6.	Roe DR, Cheatham TE. PTRAJ and CPPTRAJ: Software for Processing and Analysis of Molecular Dynamics Trajectory Data. J Chem Theory Comput. 2013;9: 3084–3095. doi:10.1021/ct400341p

7.	McGibbon RT, Beauchamp KA, Harrigan MP, Klein C, Swails JM, Hernández CX, et al. MDTraj: A Modern Open Library for the Analysis of Molecular Dynamics Trajectories. Biophys J. 2015;109: 1528–1532. doi:10.1016/j.bpj.2015.08.015
