# Supplementary material for: DyME: An MD-based engine exploiting HTP mutagenesis for protein engineering and recognition mimicry
Source: PLoS Comput Biol. 2026 Oct 7;22(10):e1014221. doi: 10.1371/journal.pcbi.1014221 (PMC13671646; doi:10.1371/journal.pcbi.1014221)
Supplement: Supplementary file 3 [file pcbi.1014221.s003.docx]

**S3 Appendix - Case Study**

**Preparation of DyME’s input files**

*- Preparation of the input structures (WT systems):* In order to model the structures needed as input (*i.e.* Abl-SH3/3BP-1 and Fyn-SH3/3BP-1 complexes), three crystal structures available at the Protein Databank were used as templates: the Abl-SH3 domain in complex with the peptide 3BP-1 (PDBID: 1ABO [1]), Abl-SH3 bound to the high-affinity peptide p41 (PDBID: 1BBZ [2]), and Fyn-SH3 in complex with the APP12 peptide (PDBID: 4ZNX [3]). Redundant chains and hetero-atoms were discarded, and the structures were superposed ensuring optimal overlap of the SH3 domains using PyMOL [4] (RMSD_Cα_ = 0.36 Å). The coordinates from peptide 3BP-1 were taken as reference wild-type peptide (peptide_WT_), and the Abl-SH3 structure from 1BBZ was selected as it has better resolution than in 1ABO (1.65 Å vs. 2.00 Å, respectively). As the atomic coordinates for the first four protein residues (MNDP) were missing, the residue numbering in the model was initially offset by four positions. In the peptide 3BP-1, amino acid R1 was removed to match the exact peptide sequence of the reference study [5]. The resulting Abl-SH3/3BP-1 and Fyn-SH3/3BP-1 complexes were saved in PDB format and prepared for MD with *pdb4amber* [6] (see Fig 2, Phase 1 on the main manuscript). Following AMBER conventions, residues were re-numbered starting from 1 for the protein and followed by the peptide, which were assigned chains A and B, respectively.

- *Upload of input structures*: Each protein/peptide_WT_ complex (from now addressed as WT systems) was loaded as input into DyME in two individual projects with identical settings. In each project, the receptor protein (Abl-SH3 or Fyn-SH3) was defined as “non-mutable object” and the ligand (3BP-1 peptide) as “mutable object”.

*- Definition of MD and scavenging parameters:* For each simulation, charges and parameters were taken from the ff19SB force field. Each system was solvated in an octahedral box of TIP3P water molecules and neutralized with Na^+^ or Cl^-^ counterions. The system was then minimized until convergence using the *minimize_energy()* function of OpenMM with default settings. Each system was heated up from 0 K to 300 K in 20 ps using the Langevin temperature coupling with a collision frequency γ = 1 ps^−1^. The system was equilibrated at constant pressure of 1 atm under periodic boundary conditions at 300 K (NPT) for 20 ps. The total simulation time was set to 2 ns per system at a temperature of 300 K. The SHAKE algorithm with a time step of 2 fs was used to constrain all bonds involving hydrogen atoms. A cutoff of 0.8 nm was applied to treat the non-bonded interactions, and the Particle Mesh Ewald (PME) method was used to treat long-range electrostatic interactions. After solvation, each system contained around ~8.600 atoms. Energy decompositions using MM-PBSA were set to use the GBSA method with ‘igb’ and “inp” values of 2 and 1, respectively (for information on these parameters, refer to the AMBER manual [7]).

- *Definition of anchor points, mutant library and clustering:* For each project, all positions of the 3BP-1 peptide_WT_ were defined as anchor points and set up for mutagenesis with a substitution matrix reproducing peptide-variants explored in the reference study [5]. As of engineering rationale, a total of ten clusters, each containing a single anchor point, were defined for DyME to generate a mutant library resembling the selected variants (*n=*32 systems, including peptide_WT_). For convenience and ease of identification, the name of each resulting mutant in the DyME database was manually set to match the naming in the reference study by repurposing the ‘type’ field on each mutant record.

**Generation of mutant structures and MD calculations by DyME**

In each project, DyME generated automatically the corresponding complex structures of each of the two proteins under study with each of the peptide-variants of the mutant library. Each protein/peptide-variant system was automatically simulated independently, using the protein/peptide_WT_ system as reference (*i.e.* 2 x 32 MD simulations).

- *Data scavenging & computing time:* DyME produced approximately 11 GB of MD trajectory data and extracted 305.788 per-residue and pairwise energy decomposition records, as well as water-site information and inter-molecular contact data. MD calculations took approximately 90 minutes using all available hardware (*vide infra*). Simulations ran in parallel using 0,25 GPU-hours each. Feature scavenging took 75 seconds per trajectory using 16 CPUs per parallel scavenger process.

*- Hardware:* The ‘Main Node and ‘Scavenger Node’ ran in a Linux server equipped with 128 AMD EPYC cores at 3.35 GHz and 512 GB of RAM. The ‘MD Node’ ran in a separate Linux server equipped with 512 GB of RAM and 10 x RTX-2080 Ti GPU cards. Both servers resided on the same Gigabit LAN. The database and project folders were hosted on a high-speed NSF partition, accessible by both servers.

**Extended Analysis of Results using the TCA**

*-* ***Overall comparative energetic analysis with the “Specificity Finder” tool:*** At first glance, given that the simulation parameters set for each project are identical, after selecting one of the projects within DyME the *“Specificity Finder”* tool allows the selection of a second project for comparative analysis. With a single mouse click, this tool generates a “*Mutant Binding Energy Comparison*” table (Fig A) containing a one-to-one comparative on protein-ligand binding energies between both projects, and with respect to their corresponding reference WT complex. This table is sortable by energy values, their differential values, or by the mutations contained per mutant. Thus, it is very useful in revealing those mutations resulting in: *i)* increased affinity with respect to WT for one or both proteins, and *ii)* specificity for either receptor (*i.e.* favorable ΔG towards Abl-SH3 while unfavorable towards Fyn-SH3). Consistent with the experimental binding measurements reported for the best engineered ligands in the reference study [5], peptides p40 and p41 (DyME’s mutant ID 29 and 30, respectively) notably emerge in DyME as top-ranked variants, illustrating the utility of DyME in effectively assessing the effects of mutations on binding affinity and specificity in a straightforward manner.

**Fig A. Illustrative example of use of DyME’s *Specificity Finder*.** Each row corresponds to the same mutant on both projects, Abl-SH3 and Fyn-SH3. ΔG values (in kcal/mol) for each mutant are listed at the column in the center, which also shows the energy difference for each mutant towards each protein receptor. A negative value represents favorable energy.

In the reference study, the dissociation constant (*K*_d_) for several peptide-variants was determined by interpolation, whereas peptide_WT_ and peptide-variants p7, p8, p17, p25, p28, p40 and p41 were measured using classical fluorescence titration[5], resulting in a higher accuracy. To assess the predictive reliability of the datasets generated by DyME, we compared the experimental log(*K*_d_) values of these high-confidence variants against their corresponding ΔG values through a linear regression(Fig B).

As shown below, the dataset for Abl-SH3/3BP-1 (Fig B1) exhibited a moderate-to-strong linear fit and a strong Pearson correlation (R² = 0.76, r = 0.87). The dataset for Fyn-SH3/3BP-1 (Fig B2) resulted in similar fitness values (R² = 0.65, r = 0.81), indicating that both datasets offer adequate baseline levels of predictive utility, as seen in the two best peptides (p40 and p41) and other variants properly ranked by DyME and in complete agreement with experimental results.

**Fig B. Scatter plots of log(*K*_d_) versus ΔG for Abl-SH3/3BP-1** **(1) and Fyn-SH3/3BP-1 (2).** Each dot corresponds to a mutant. Linear regression lines are shown in red. Pearson correlation coefficients and R^2^ values are reported in the table at the top of each graph. Peptide-variants p40 and p41 are framed in a green square in both plots for ease of identification.

To further exemplify the utility of DyME in guiding a design rationale, in Table A, we illustratively compare the ΔG values obtained with DyME and the experimental binding data from the reference study [5] for the 2 best variants (peptides p40 and p41) and four other high-confidence variants (*i.e.* p8, p17, p25 and p28). In accordance with the design strategy of the reference study [5], the peptide-variants reported to have the best *K*_d_ values towards Abl-SH3 also show the most favorable predicted binding energies in DyME. Likewise, peptide_WT_ (3BP-1) and those variants reported to have high *K*_d_ values for Abl-SH3 (*i.e.* p25) are predicted by DyME to have less favorable binding energy values.

**Table A.** **DyME’s binding energy (ΔG) values versus experimentally obtained *K_d_* values** [5]

| **LIGAND** | | **LIGAND-RECEPTOR EXPERIMENTAL *K*_d_ AND BINDING ENERGIES** | | | | | |
| --- | --- | --- | --- | --- | --- | --- | --- |
|  |  | **Abl-SH3** | | | **Fyn-SH3** | | |
| **Name** | **Sequence^(a)^** | **Mutant ID**^(b)^ | ***K*_d_ (μM)**^(c)^ | **ΔG (kcal/mol)**^(d)^ | **Mutant ID**^(b)^ | ***K*_d_ (μM)**^(c)^ | **ΔG (kcal/mol) )**^(d)^ |
| **3BP-1** | RAPTMPPPLPP | 1 | 34 ± 3 | -27,5 ± 3,7 | 1 | 34 ±3 | -26,0 ± 2,4 |
| **p8** | -APT**Y**PPP**A**PP | 7 | 5 ±0,6 | -28,3 ± 4,7 | 7 | 65 ±3 | -22,4 ± 2,8 |
| **p17** | -APT**YS**PPLPP | 16 | 2 ± 0,3 | -28,6 ± 2,9 | 16 | 136 | -26,7 ± 3,8 |
| **p25** | -A**FTY**PPPLPP | 24 | 41 | -24,5 ± 2,8 | 24 | 161 | -23,5 ± 3,4 |
| **p28** | -**FG**T**Y**PPPLPP | 27 | 8 ±10 | -29,2 ± 3,5 | 27 | 97 ±10 | -23,0 ± 2,4 |
| **p40** | -APT**YS**PP**P**PP | 29 | 0,4 ±0,1 | -31,8 ± 4,8 | 29 | 472 ±55 | -16,8 ± 3,61 |
| **p41** | -AP**SYS**PP**P**PP | 30 | 1,5 ±0,1 | -30,7 ± 3,3 | 30 | 273 ±30 | -18,8 ± 3,3 |

^(a)^ Ligand sequence. Point mutations with respect to peptide_WT_ 3BP-1 are highlighted in red. ^(b)^ Internal mutant ID assigned during the DyME workflow for ease of identification in the DyME-TCA.^(c)^ Experimental *K*_d_ values from reference study[5].^(d)^ Predicted binding energies obtained from DyME.

*-* ***In-depth comparative analysis with DyME-TCA****:* This toolbox enables a thorough examination of the obtained MD-based data. It facilitates both visual and numerical in-depth exploration in an intuitive and effective manner. An exploration session was initiated through the “*Mutant Explorer*” tool and the *“Mutant Summary”table*. The widgets available for comparative analysis of the results obtained in project Abl-SH3/3BP-1 are summarized in Fig C. In particular, the screenshot shown as example contains a detailed comparative of peptide_WT_ and variants p25, p28, p40, p41(DyME’s ID 24, 27, 29 and 30, respectively). Data on any other desired variant to be analyzed can be simply added in a cumulative fashion at the user’s convenience with a single click. Likewise, mutants can be filtered by selecting the corresponding *“Advanced Filter”* option.

**Fig C. Example of comparative analysis of results from project Abl-SH3/3BP-1 using the DyME-TCA.** At the labels, under the graphs, peptide_WT_ is shown in grey, and variants p25 (ID 24), p28 (ID 27), p40 (ID 29) and p41 (ID 30) are shown in red, green, yellow and blue, respectively.

The *“Mutant Simulation Results”* panel (Fig C1) displays a statistical and numerical overview of the binding energies obtained for each entry in the mutant library for project Abl-SH3/3BP-1. At the top, the “*Binding Energy Threshold*” widget shows the baseline energy (-27,5 kcal/mol) as established by the WT complex, the worst energy for mutant p25 and the best value for mutant p40 (-24.5 and -31,8 kcal/mol; respectively). Below, the “ΔG Percentile” classification of the *“Statistical Summary Data”* widget helps the user to visualize clearly that at least 50% of all mutants achieved favorable energy gains with respect to WT. Those appear conveniently highlighted in green on the *“Mutant Summary”* table underneath. In agreement with the reference study [5], variants p40 and p41 (DyME’s IDs 29 and 30, respectively) appear at the top of the table with the most favorable binding energies. Likewise, variant p25 appears at the bottom of the table, indicating the less favorable binding energy of the mutant library.

The “*RMSD Explorer*” widget (Fig C2) at the *“Data Viewer Panel”* generates an interactive plot that helps the user investigate simulation convergence (*i.e.* in our example case, convergence after 400ps) and if mean displacement values remained within acceptable values (*i.e.* in this case 3.10 to 3.30 Å).

The “*Per-residue Energy at Anchor points*” interactive plot generated by DyME (Fig C3) facilitates a straightforward energetic comparison between mutations, highlighting differential energy gain/loss of the selected mutant-variants with respect to WT. Note: In this specific case, the anchor point labels have been toggled for comparative convenience, translating residue numbers from 1-10 in the reference study [5] to 59-68, to match those in the X-ray crystal structures used for creating the models of the WT systems. For a more detailed analysis, an expanded view of this widget is provided in Fig D.

**Fig D. *Per-residue Energy at Anchor points* widget from project Abl-SH3/3BP-1.** The Y-axis represents ΔG (in kcal/mol). The X-axis corresponds to each anchor point. Labels at bottom indicate the mutant ID and color assigned to each variant being explored. Each bar represents per-residue energy at a given position and the error bars represent the standard error of the mean, as reported by MM-GBSA. The residue name at each position is indicated on top of each bar as a 1-letter code in red.

The per-residue energy values overview offered by this widget allows a broad energetic assessment of each mutation on all variants being explored, with respect to WT. For instance, peptide residues A59 and P60 (corresponding to positions 1 and 2 in the peptide sequence numbering of the reference study [5]) were mutated to F59 and G60 in variant p28 (ID 27) to test whether the introduction of flexibility through a glycine in the sequence could allow the aromatic ring of Phe at position 59 to pack more favorably with the surrounding aromatic residues of Abl-SH3 [5]. Variant p25 (DyME’s ID 24), with the combination A59 and F60, was used as control. The obtained results suggested favorable energy for Phe at position 1 in contrast to Ala, which showed unfavorable binding energy. Interestingly, while Phe exhibited a favorable binding energy at position 2, its interacting energy was still lower than that for Pro. Furthermore, Gly at position 2 appeared irrelevant for binding.

Thus, in agreement with the experimental data from the reference study [5], this plot facilitates the straightforward identification of a 40% and 100% change in energy at position 60 with respect to peptide_WT_ for mutants p25 and p28, respectively, and a distinctive shift from unfavorable to favorable energy in position 59 of mutant p28. This prediction by DyME aligns with the hypothesis suggested by the authors of the reference study based on the relatively good fitness of this variant, despite losing all contacts at position 60.

A clearer example of DyME’s utility resides in its predictions at positions 61, 63 and 66 (corresponding to 3, 5 and 8 in the peptide sequence numbering of the reference study [5]). A closer look at variant p40 (ID 29) and p41 (ID 30) shows how mutations P63S and L66P in both variants, and T61S in variant p41, are predicted to outperform the energetics of the WT residue at those positions.

The *“Interactive Energy Explorer”* widget (Fig C4) visualizes the energetic impact of ligand mutations from the perspective of the protein receptor (*i.e.* *“non-mutable object”*) in a sequence- or position-based comparative manner. DyME greatly facilitates this analysis using a color gradient that highlights energy variations at each position using peptide_WT_ as baseline reference. For a more detailed examination, an expanded view of this widget is provided in Fig E.

**Fig E. *Interactive Energy Explorer* widget presenting the Energy Gain/Loss perspective on variants in the exploration session (peptide_WT_, p25, p28, p40 and p41) of the Abl-SH3 project.** The sequences of the mutable objects (peptides) are shown on the left side, preceded by their respective internal mutant ID assigned by DyME and their given name in the reference study. The sequence of the protein receptor, Abl-SH3, is shown on the right. The sequence at the top corresponds to the Abl-SH3 in complex with peptide_WT_, and colors blue and red are used to indicate stabilizing and destabilizing energetic contributions, respectively. All other sequences show energy gains or losses with respect to Abl-SH3/peptide_WT_ using a color gradient from green to red, respectively.

The visual option “Energy gain/loss” offered to the user in this interactive widget clearly highlights significant energy fluctuations across the variants selected for analysis in comparison to the WT, while revealing straightforward which interfacial residues make intermolecular contact, and, at each position, whether these contacts are more favorable or unfavorable. In this particular example, mutant-variants p40 and p41 are predicted to exhibit pronounced binding energy gains from the perspective of their interaction with the protein receptor (Abl-SH3) at three regions: residues 7–16, 31–36, and 47–52. This tool not only pin-points the regions of interaction with the protein receptor and responsible for energetic changes, but it also captures the detailed energetic profiles unique to each peptide-variant. Notably, the residues identified by DyME are in agreement with those discussed in the reference study [5]. While variants p40 and p41 display the most favorable interactions, variant p25 is predicted a marked trend toward less favorable interactions relative to WT, consistent with experimental observations. These results clearly demonstrate the particular advantage of using this tool to discriminate at first glance variants and mutations with predicted enhanced/reduced recognition, assisting the user to easily and rapidly select the best variants saving on experimental efforts.

The “*Pairwise Contact & Energy*” (Fig C5) allows the user to interactively, and on-demand, further explore the effect of mutating positions through a pairwise energy decomposition map showing the specific values of each anchor point contacting one or more residues at the receptor. In this widget, pairwise contacts formed by each mutant are shown as squares following the corresponding color code. For this particular example, known interactions achieved with the introduction of mutations in peptide_WT_ 3BP-1 to obtain variants p40 and p41, and reported in the reference study, are successfully scavenged and shown by DyME (see “mutant-unique contacts” circled in red in Fig C5). For instance, the authors of the reference study found that Ser is better than Pro at position 5 in the peptide sequence (corresponding to position 63 in the protein/peptide complex), given that it could potentially make a strong hydrogen bond to the side chain of E35 in Abl-SH3 (E38 in the reference study). This position was reported to play a key role in increased specificity [5]. DyME conveniently highlights this important contact and reveals a favorable energy shift from 0.54 to -3.51 kcal/mol when using Ser, which improves significantly with respect to the wildtype residue (Pro) at this position. Showcasing a similar example, the widget reveals that Thr at position 3 (numbering of the reference study) of the peptide (61 in the protein/peptide complex) forms a hydrogen bond of –2.33 kcal/mol with Asn in position 33 at the protein receptor (36 in the reference study) in variant p40, but not in variant p41, which mutates for a Ser at this position. Numerous mutant-unique emerging contacts can be easily identified for peptide-variant p40 (circled in red in Fig C5) and also strongly conserved contacts (circled in green in Fig C5), consistent with mutation Met to Tyr in position 4 of the peptide (position 62 in the complex), which occurs in all mutant-variants selected for exploration, demonstrating the power of the TCA in scrutinizing pairwise contact data.

DyME allows the user to perform an intuitive exploration in a quite straightforward manner, and it is particularly useful for elucidating the emergence of mutant-specific contacts with ease. The energy thresholding feature of this widget makes it easy to expose the relative strength of pairwise contacts between variants and discern weak from strong interactions among all peptide-variants being explored.

The *“Interface Contact Explorer”* widget (Fig F) helps the user to comprehensively analyze contact information and rationalize the emergence of novel or altered contacts across the investigated systems. The contact table provided by this widget (Fig F1) displays mutant-specific interactions that are either conserved or altered relative to WT, providing a concise overview of mutation-induced changes in interfacial contacts at glance. The number of contacts at each position are shown in green cells when identical to the WT, and in red cells when differing. To showcase a representative example using the 10% threshold filter, intermolecular contact details are provided at atom-level by clicking the row of interest (Fig F2).

The Contact Explorer table (Fig F1) can conveniently be used in tandem with the pairwise energetic decomposition map (Fig C5) to obtain detailed energetic insights on newly emerging contacts. In this particular example shown, the user can observe that mutation P63S (P3S in the reference study) of peptide p40 is favorable, as the side-chain hydroxyl oxygen (OG) of Ser is predicted to form hydrogen bond with both carboxylate oxygens (OE1 and OE2) of residue E35 in the protein receptor. Additionally, the serine beta-carbon (CB) is predicted to form van der Waals interactions with the delta-carbon (CD) of E35. Likewise, Fig F3 illustrates an example of combining interface contact data with pairwise energy data obtained from the “*Pairwise Contact & Energy*” widget for peptide p40 between T61 and E35, and between S63 and E35, revealing this potentially favorable mutations while reporting its estimated energetic contribution (Fig F4), at just a mouse click. This way, the user is easily made aware that the mutation P63S in p40 improves contact to E35 with respect to WT.

**Fig F. Interface Contact Explorer widget for project Abl1-SH3/3BP-1.** (1) Each column represents a peptide-variant selected for investigation. Rows indicate positions predicted to form inter-molecular contacts with the protein receptor and shown in red if the position corresponds to an anchor point. Table cells are colored red and green to indicate whether the number of intermolecular contacts differs from that of the WT or remains the same, respectively. (2) Tooltip illustrating detailed contact data for mutation P63S in peptide-variant p40 with residue E35 at the protein receptor. (3) Comparative example with data from the Pairwise Contact & Energy widget (from C5 Fig) showing the energy of such emerging contact (4).

The *“Water Site Explorer Widget”* (Fig G) included in DyME allows the user to examine water-sites and water-mediated contacts obtained from MD trajectories in a very straightforward and user-friendly fashion*.* Moreover, it enables the user to do so simultaneously on all variants selected for exploration and easily determine whether water-sites are conserved or altered with certain point-mutations, irrespective of water molecules having different IDs across simulations. As an example to illustrate these capabilities, we also use the Abl-SH3/3BP-1 project, as water-mediated interactions have been reported to be relevant for recognition of the improved p41 peptide-variant [8]. For comparative purposes, we have used data obtained from follow-up studies revealing the high resolution crystal structure of the Abl-SH3/p41 complex [2] and additional thermodynamic studies investigating the role of the discovered interfacial crystallographic waters [9].

Using the data obtained from the MD simulations performed by DyME for peptide p41 (DyME’s mutant ID 30), and in comparison, to those for peptide_WT_ (ID 1) and the other selected peptide-variants, within the Water Site Explorer widget (Fig G1) the user can visualize a report on the number of interfacial water-sites identified (9 in this case) and the number of water molecules involved (a total of 21) based on their position and residence time.

A click on the *“View Waters”* button outputs the *“Water-contacts Summary Panel”* (Fig G2), an interactive table that facilitates a deeper inspection of the water molecules involved in the formation of a specific water site being selected for analysis. Likewise, this table facilitates the identification of those water molecules residing near more than one residue, indicative of a possible role in water-mediated interactions, as well as their combined residence near interfacial residues. Notably, in this particular example with variant p41, the majority of water-sites identified by DyME matched closely those reported in the follow-up experimental study [8].

The interactive function built into this table allows hovering on a particular water molecule to easily visualize its proximity to other residues. For instance, in our specific example with p41, the water molecule with ID 8515 is observed residing near residue D14 at the receptor for 19% of the simulation time, and A59 and P60 (positions 1 and 2 in the sequence numbering for peptide p41) for 100% of the simulation time. Likewise, water ID 2716 resides by receptor residue N51 for 41% of the simulation time and simultaneously near P64 and P65 (positions 5 and 7 in the reference study) for 19% of the simulation time, indicating possible water-mediated interactions.

This interactive table is linked to the “3D Explorer Widget”, which automatically visualizes the centroids of identified water-sites as spheres (Fig G3) overlapping with those identified in the peptide_WT_ and other variants being explored (p41 in this case), providing straightforward spatial superposition of water-sites for analysis. Each centroid includes a label indicating its residence time. The “Display Control” panel at the left of the 3D viewer provides the means to toggle water-sites on/off for any variant. This tool showcases DyME’s capabilities in terms of comparative water-site display across mutants selected for exploration as well as the effectiveness of DyME in successfully identifying water-sites in agreement with experimental data, and the effortless capability of the built-in *“Water Site Explorer”* widget for in-depth investigation in a user-friendly intuitive manner.

\

**Fig G. Water Site Explorer widget for project Abl1-SH3/3BP-1.** (1) Table containing a count of sites as identified by DyME for WT, p41 and other mutant-variants. (2) Water-contacts Summary Panel for peptide p41 (mutant ID 30). (3) 3D Explorer widget displaying water-site centroids of WT (grey) and peptide p41 (blue), as well as the complex structures of Abl-SH3 with WT (grey ribbons) and p41 (blue ribbons) peptides.

**References**

1.	Musacchio A, Saraste M, Wilmanns M. High-resolution crystal structures of tyrosine kinase SH3 domains complexed with proline-rich peptides. Nat Struct Mol Biol. 1994;1: 546–551. doi:10.1038/nsb0894-546

2.	Pisabarro MT, Serrano L, Wilmanns M. Crystal structure of the abl-SH3 domain complexed with a designed high-affinity peptide ligand: implications for SH3-ligand interactions. J Mol Biol. 1998;281: 513–521. doi:10.1006/jmbi.1998.1932

3.	Camara-Artigas A, Ortiz-Salmeron E, Andujar-Sánchez M, Bacarizo J, Martin-Garcia JM. The role of water molecules in the binding of class I and II peptides to the SH3 domain of the Fyn tyrosine kinase. Acta Cryst F. 2016;72: 707–712. doi:10.1107/S2053230X16012310

4.	The PyMOL Molecular Graphics System, Version 1.3r1 | Ncdirtrd3 | Zotero. [cited 30 Jan 2025]. Available: https://www.zotero.org/groups/167781/ncdirtrd/items/HJ2H2FHT/item-details

5.	Pisabarro MT, Serrano L. Rational Design of Specific High-Affinity Peptide Ligands for the Abl-SH3 Domain. Biochemistry. 1996;35: 10634–10640. doi:10.1021/bi960203t

6.	Case DA, Aktulga HM, Belfon K, Cerutti DS, Cisneros GA, Cruzeiro VWD, et al. AmberTools. J Chem Inf Model. 2023;63: 6183–6191. doi:10.1021/acs.jcim.3c01153

7.	Case DA, Aktulga HM, Belfon K, Ben-Shalom IY, Berryman JT, Brozell SR, et al. Amber 2022 Reference Manual. University of California, San Francisco; 2022. Available: https://ambermd.org

8.	Palencia A, Camara-Artigas A, Pisabarro MT, Martinez JC, Luque I. Role of interfacial water molecules in proline-rich ligand recognition by the Src homology 3 domain of Abl. J Biol Chem. 2010;285: 2823–2833. doi:10.1074/jbc.M109.048033

9.	Palencia A, Cobos ES, Mateo PL, Martínez JC, Luque I. Thermodynamic dissection of the binding energetics of proline-rich peptides to the Abl-SH3 domain: implications for rational ligand design. J Mol Biol. 2004;336: 527–537. doi:10.1016/j.jmb.2003.12.030
