# Supplementary material for: DyME: An MD-based engine exploiting HTP mutagenesis for protein engineering and recognition mimicry
Source: PLoS Comput Biol. 2026 Oct 7;22(10):e1014221. doi: 10.1371/journal.pcbi.1014221 (PMC13671646; doi:10.1371/journal.pcbi.1014221)
Supplement: Supplementary file 4 [file pcbi.1014221.s004.docx]

**S4 Appendix – Installation and MD Test Data**

**Working with the DyME test data**

- The test data of the case study contains 64 MD simulations produced by DyME, as well as the scavenged records allocated in their corresponding database collections, which the installer can load automatically if chosen by the user.
- Simulation settings and parameters can be found in S3 Appendix (Case Study), as well as a detailed explanation on how DyME facilitates comparative analysis of MD-data.
- The inputs, outputs and database records have been deposited on Zenodo [1] (<https://zenodo.org/records/18014320>), hosting a single tar file “dyme-test-data-md.tar”, and a short README file describing its contents. The README can be accessed by opening the above link in any browser.
- It is not necessary to “manually” download or deposit this file onto the platform.
- The installer contains scripts that obtain and deposit the test data automatically in appropriate folders and database collections. The test data can be explored from the DyME TCA or accessed manually in the /project subfolder of the dyme_root main folder (set during installation).
- The MD settings, molecular maps, clusters, and anchorpoint definition data for each project are available on the database collection “projects” under project_id’s 49 and 50.
- Only the Main Node is required to run to explore test data (it provides the TCA, database, and API services). Worker nodes are not required to use the DyME-TCA or explore existing results.
- Once the main node is running and test data has been deposited, the DyME database can also be accessed using “MongoDB Compass” or any other suitable database client using the following connection string:

**mongodb://hostname_of_dyme_main_node:27017/**

**Quick Installation Instructions**

For detailed information on installing the platform and running the Main Node, refer to the README file at the Github repository (<https://github.com/pisabarro-group/DYME>)

**References**

1.	Guillem Gloria P manuel. DyME Test Data - Raw MD inputs and outputs. Pedro Manuel Guillem-Gloria, Gloria Ruiz-Gomez, M.Teresa Pisabarro; 2025. doi:10.5281/zenodo.18014320
